# Dysregulation of SRSF11 in Cancer: Mechanistic Insights and Biomarker Potential for Diagnosis and Therapy

**DOI:** 10.7150/jca.123791

**Published:** 2026-01-14

**Authors:** Yang Jun, Liu Shanshan, Xiao Jingwen, He Yu, Xiao Jianlong, Shi Qingfeng

**Affiliations:** 1 Department of Laboratory Medicine, Guilin people's Hospital, No.12 Wenming Road, Guilin, Guangxi, China, 541002.; 2 Department of Laboratory Medicine, Affiliated Hospital of Guilin Medical University, No.15 Lequn Road, Guilin, Guangxi, China, 541001.; 3 School of Biology, Faculty of Applied Sciences, Universiti Teknologi Mara, UiTM, 40450, Section 2, Shah Alam, Selangor, Malaysia.

**Keywords:** SRSF11, alternative splicing, telomerase, EMT, biomarkers, splicing therapy

## Abstract

Serine/arginine-rich splicing factor 11 (SRSF11) is an RNA-binding regulator that modulates alternative splicing and RNA metabolism in a context-dependent manner across selected malignancies. Evidence from colorectal, hepatocellular, gastric, glioma, and a few other cancers indicates that SRSF11 participates in cell-cycle regulation, telomerase recruitment, and epithelial-mesenchymal transition (EMT) through specific signaling axes, including PAK5-SRSF11-HSPA12A in colorectal cancer, METTL3-SRSF11 in gastric and breast cancers, and SRSF11-CDK1/telomerase circuits in hepatocellular carcinoma. These mechanisms highlight SRSF11 as a candidate biomarker for diagnosis and prognosis rather than a universal oncogenic driver. We summarize the current mechanistic, post-translational, and non-coding RNA-mediated regulatory evidence, clarify the limitations of existing data, and propose future multi-omics and functional approaches to validate SRSF11-directed splicing therapy. This review integrates mechanistic insight with clinical evidence while emphasizing cancer-specific rather than generalized conclusions.

## Introduction

The dysregulation of Precursor messenger RNAs (pre-mRNAs) splicing, which involves the precise removal of introns and joining of exons to generate mature mRNAs, has emerged as a hallmark of cancer, profoundly influencing the initiation and progression of malignancies^1^, ^2^. Among the splicing regulators, serine/arginine-rich splicing factor 11 (SRSF11)—a member of the SR protein family—has attracted growing attention for its roles in pre-mRNA processing, RNA metabolism, and cellular homeostasis^3^. Increasing evidence suggests that aberrant SRSF11 expression contributes to tumorigenesis by altering splicing programs that affect cell proliferation, survival, and migration^4^-^6^. However, these findings are supported primarily in specific cancer types, such as colorectal cancer (CRC), hepatocellular carcinoma (HCC), gastric cancer, and glioma, whereas data in other tumors (e.g., ovarian, breast, prostate) remain limited or correlative. Despite substantial progress in understanding SRSF11-mediated splicing regulation, the mechanisms underlying its dysregulation and cancer-type specificity remain incompletely defined. For example, mutations in RNA-binding motifs or post-translational modifications may alter its splicing activity, generating oncogenic isoforms, yet direct experimental evidence for these processes is still emerging. Moreover, how SRSF11 integrates with upstream signaling cascades or whether its modulation could be exploited therapeutically to target cancer-specific splicing events warrants further investigation.

pre-mRNAs, the direct byproducts of transcription, contain non-coding introns interspersed among distinct exons^7^. Before these pre-mRNAs can be converted into mature mRNAs, they must undergo multiple processing modifications^8^. Unlike constitutive splicing, which consistently removes introns, alternative splicing (AS) selectively determines exon inclusion or exclusion^9^, ^10^., generating multiple transcript isoforms from a single gene and allowing cells to adapt to diverse physiological conditions. AS is estimated to affect over 95% of human genes^11^. In cancer, splicing dysregulation often produces tumor-specific isoforms that enhance malignant traits such as sustained proliferation, immune evasion, increased motility, and therapeutic resistance^12^.

The SR protein family represents a conserved group of RNA-binding proteins that serve as key regulators of AS^13^. 12 SR family members (SRSF1-SRSF12) and two SR-like proteins, TRA2A and TRA2B, have been identified in human cells^14^. TRA2 proteins, characterized by a single RNA recognition motif (RRM) and two RS domains, function as sequence-specific splicing activators^15^. SR proteins are crucial for regulating gene expression, participating in processes such as constitutive and alternative splicing of pre-mRNA, as well as mRNA nuclear export, stability, and translation^16^. In recent years, many SR protein family members have been found to exhibit aberrant expression in various tumors. For example, Wan et al.^17^ demonstrated that SRSF6 induces abnormal splicing of the tight junction protein ZO-1, promoting colorectal cancer progression. Similarly, SRSF9 regulates apoptosis by targeting caspase-2 to produce two functionally distinct splicing isoforms^18^, while SRSF3 is downregulated in liver cancer tissues and cells, suggesting a potential tumor-suppressive role^19^. Despite extensive studies on SR proteins, the regulatory mechanisms driving SRSF11's cancer-specific roles remain unclear.

SRSF11 plays multiple roles in RNA processing and its dysregulation contributes to carcinogenesis^20^. Under normal cellular conditions, SRSF11 is essential in regulating alternative splicing, ensuring accurate mRNA processing and transcriptomic integrity^21^, ^22^. In cancer, SRSF11 overexpression has been demonstrated in a limited number of malignancies, including CRC, HCC, gastric cancer, and glioma, where it regulates processes such as cell-cycle progression, telomerase activation, epithelial-mesenchymal transition (EMT), and drug resistance^23^, ^24^. In contrast, studies in other cancers remain preliminary or correlative. Aberrant SRSF11 activity can generate oncogenic splice variants that enhance tumorigenic potential, although these effects appear context-dependent rather than universal. Collectively, these findings highlight SRSF11 as a promising but cancer-specific biomarker and therapeutic target, meriting further validation in broader tumor types.

This review aims to provide a comprehensive overview of SRSF11 dysregulation in cancer, focusing on its mechanistic roles in splicing regulation, RNA metabolism, and tumor biology. We summarize established evidence from well-characterized cancers, identify gaps in current knowledge, and discuss the potential diagnostic, prognostic, and therapeutic implications of targeting SRSF11 in a context-dependent manner.

## Structure and Function of SRSF11

**Figure 1:**
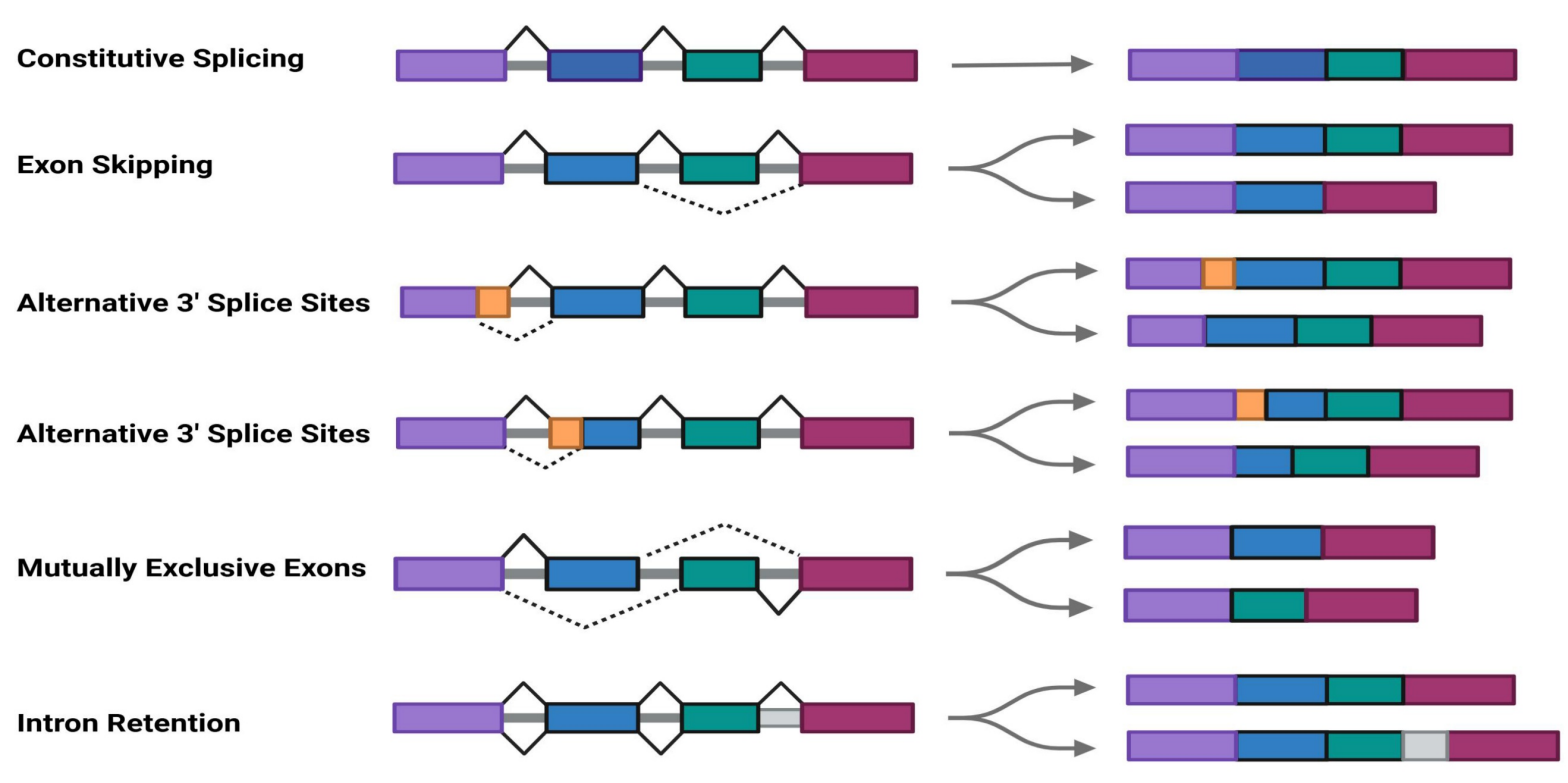
Schematic motif of SRSF11 structure and alternative splicing. A. SR family and their domain structure.B. SRSF11 structure and phosphorylation domain of SRPK and CLK.C. Schematic depiction of constitutive splicing and five modes of alternative splicing: exon skipping (ES), alternative 5' splice sites (A5' SS), alternative 3' splice sites (A3' SS), intron retention (IR), and mutually exclusive exons (MXE).

The SRSF11 gene is located on chromosome 1p31.1 and encodes a protein of 484 amino acids (AA) with a molecular weight of 54 kDa^14^. It has two key domains: an RNA recognition motif (RRM) for binding specific RNA sequences and a serine-arginine-rich (SR) domain for protein-protein interactions^25^ (Fig. 1A). The RRM enables SRSF11 to recognize the exonic splicing enhancer (ESE) in precursor mRNA^4^, ^26^, whereas the SR domain facilitates recruitment of spliceosomal components and assembly of functional splice complexes^27^ (Fig. 1B). This dual-domain architecture allows SRSF11 to act as an RNA-binding regulatory factor that modulates spliceosome assembly, rather than serving as a core structural component of the spliceosome^28^. Alternative splicing of SRSF11 can produce variants lacking portions of the RRM or RS domains, resulting in functionally distinct isoforms. Eight transcript variants have been reported, some (e.g., UC009) lacking the RS region required for full splicing activity^29^. SRSF11 is predominantly localized in nuclear speckles, where it coordinates splice-site selection and RNA export^20^. Its activity and localization are dynamically regulated by post-translational modifications, particularly phosphorylation, allowing the protein to fine-tune splicing regulation in response to cellular conditions^30^.

## Role of SRSF11 in RNA Splicing

pre-mRNAs contain introns that must be removed during splicing to produce mature mRNAs^31^. Through alternative splicing , pre-mRNAs can generate diverse isoforms to meet specific cellular or environmental demands^28^, ^32^. SRSF11 functions as a trans-acting regulatory factor involved in spliceosome assembly and splicing reactions, promoting pre-mRNA splicing and contributing to various AS patterns, including exon skipping (ES), alternative 5' splice sites (A5' SS), alternative 3' splice sites (A3' SS), intron retention (IR), and mutually exclusive exons (MXE)^33^(Fig.1C).

SRSF11 is enriched in nuclear speckles, which are hubs for splicing factors, and it plays a crucial role in telomerase regulation through the telomerase RNA component (TERC)^20^. It stabilizes the telomerase-telomere complex, enhances telomerase activity, and regulates telomerase localization^34^. During the S phase of the cell cycle, SRSF11 binds to TERC and facilitates telomerase recruitment to nuclear speckles, ultimately promoting telomere elongation^35^, ^36^. Loss of SRSF11 disrupts telomerase localization, impairing telomere maintenance and contributing to genomic instability^20^. These features position SRSF11 as a potential therapeutic target for controlling telomerase activity in cancer cells.

In addition to its oncological relevance, SRSF11 has also been identified as a splicing suppressor of Tau gene exon 10[37]and as a regulator of exon 7 inclusion in the SMN1 gene^38^, ^39^. However, these findings are derived from neurological and neuromuscular disease models rather than cancer systems, and are presented here solely to illustrate the mechanistic versatility of SRSF11 in RNA processing.

Beyond its splicing functions, SRSF11 also contributes to RNA stability, nuclear export, and translation, underscoring its multifunctional role in RNA metabolism^40^. Recent studies[3, 41]further indicate that loss of SRSF11 alters the splicing of LRP8 and ApoE, inactivating the JNK signaling pathway and impacting aging. These findings suggest that SRSF11 may influence age-related biological processes by regulating ApoE and LRP8 expression.

## Regulation of SRSF11 Expression and Function

SRSF11 plays a key role in alternative splicing, allowing a single gene to produce multiple transcript isoforms^42^. This function is essential for maintaining the diversity and adaptability of the cellular proteome^43^. By binding to exon or intron splicing enhancers, SRSF11 controls splicing site selection, enabling the inclusion or exclusion of specific exons^44^. Proper regulation of SRSF11 expression and activity is essential for tissue-specific splicing and cellular responses to environmental signals.

Recent studies indicate that SRSF11 expression and activity are tightly regulated to produce alternative splicing products involved in key cellular processes, such as cell cycle progression, apoptosis, and differentiation^4^, ^5^, ^45^. These regulatory mechanisms include post-translational modifications(PTMs) such as phosphorylation, methylation, acetylation, and ubiquitination, as well as protein-protein interactions and regulation by non-coding RNAs. While phosphorylation is a well-recognized regulatory mechanism among SR family proteins, the specific phosphorylation sites and functional consequences for SRSF11 remain only partially characterized. Some evidence regarding phosphorylation-dependent control of subnuclear localization and splicing activity is extrapolated from studies of related SR proteins (e.g., SRSF1 and SRSF7), and further direct validation for SRSF11 is warranted.

**Figure 2:**
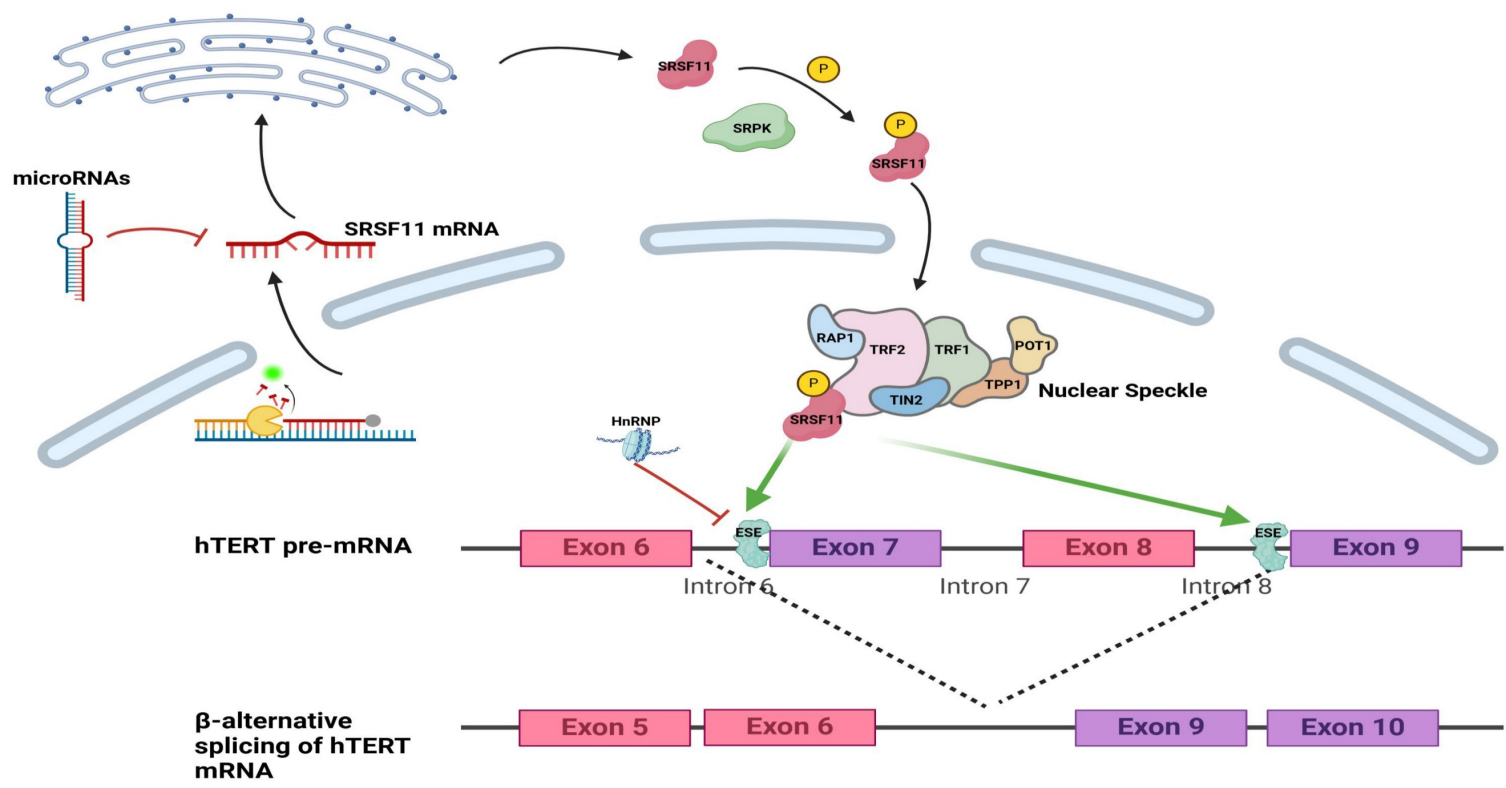
Schematic presentation of SRSF11 and regulatory factor in alternative splicing of hTERT pre-mRNA. A. SRSF11 mRNA entered the cytoplasm and was regulated by miRNAs and METTL3. B. The RS domain of the SRSF11 protein is phosphorylated under the action of SRPK. C.SRSF11, as a critical telomere recognition unit, is incorporated into nuclear speckles. D.SRSF11 competes with HnRNP to recognize the ESE sites on exon 7 and exon 9 of hTERT, promoting the generation of the β-transcript variant of hTERT mRNA. ESE: exonic splicing enhancer.

In cancer, abnormal regulation of SRSF11 alters splicing events, generating isoforms that promote tumor development^22^, ^25^. Moreover, SRSF11 influences telomerase activity by modulating alternative splicing of human telomerase reverse transcriptase (hTERT), a key enzyme responsible for maintaining chromosomal ends. Through this mechanism, SRSF11 participates in oncogenic transformation, embryonic development, and stem cell differentiation^46^ (Fig. 2). This underscores the central importance of SRSF11 in coordinating splicing regulation and highlight its potential as a context-dependent therapeutic target in oncology.

### Phosphorylation: Regulation of SRSF11 Function and Location

SRSF-related kinases play a key role in regulating the function of SRSF11. SR proteins, including SRSF11, undergo phosphorylation and dephosphorylation cycles by various protein kinases, leading to changes in their subcellular localization and activity^6^. These kinases, closely linked to AS events, are collectively referred to as splicing-specific kinases or SR protein-related kinases^25^. Key members of this group include SR protein kinases(SRPKs) and Cdc2-like kinases(CLKs)^31^. The phosphorylation state of SRSF11 controls its movement between the nucleus and cytoplasm, influencing its roles in nuclear splicing and cytoplasmic functions, such as RNA transport and stability^47^. In the cytoplasm, SRSF11 is phosphorylated by SRPKs, which is a prerequisite for its nuclear import via transportin-SR2 (TRN-SR2)^31^. Unlike SRPKs, which facilitate nuclear import, CLKs are mainly found in the nucleus, where they regulate the distribution of SR proteins through phosphorylation^6^.

SRSF11 activity is modulated by the phosphorylation of serine and arginine residues within its SR domain^48^. SR protein kinases, such as SRPKs and CLK kinases, play a central role in this process^4^. Phosphorylation not only influences SRSF11's subcellular localization but also affects its interactions with other splicing factors and its splicing activity^22^.

### Regulation by Transcription Factors

The expression of SRSF11 is also regulated at the transcriptional level by transcription factors that respond to cellular signals^49^. For instance, SRSF11 has been linked to the regulation of erythroid and granulocyte differentiation^50^. In CD34^+^ hematopoietic stem and progenitor cells (HSPCs), the knockdown of SRSF11 results in upregulating L-ELK1 expression. This suggests that SRSF11 may influence erythroid and granulocyte differentiation by modulating the splicing of ELK1^51^. Moreover, regulatory elements in the SRSF11 promoter region respond to stimuli such as hypoxia, growth factors, and oncogenic signals, resulting in dynamic changes in SRSF11 expression levels^52^, ^53^.

### Regulation by Non-Coding RNAs

SR proteins, including SRSF11, interact with non-coding RNAs (ncRNAs) that are critical in regulating gene expression and splicing activity^54^. These ncRNAs include small nucleolar RNAs (snoRNAs), microRNAs (miRNAs), and long non-coding RNAs (lncRNAs), each contributing to distinct regulatory pathways^55^.

miRNAs can target the mRNA of SRSF11 for degradation or translational repression^56^. In particular, cancer-associated miRNAs may downregulate SRSF11, altering its role in alternative splicing and influencing cancer progression^33^. For example, a study on primary acute myeloid leukemia (pAML) identified hsa-miR-133 was significantly downregulated and regulated ZC3H15, BCLAF1, SRSF11, KTN1, PRPF40A, andGNL2^57^. This finding highlights the potential role of miRNAs in modulating SRSF11 expression and its downstream effects on splicing regulation and cellular processes.

lncRNAs also contribute to the regulation of SRSF11. For instance, lncRNA HOTAIRM1 was predicted to regulate SRSF11 through a computational model integrating protein-lncRNA heterogeneous networks, diffusion features, HeteSim features, and a gradient boosting tree (GTB) algorithm^58^. However, this regulatory relationship remains to be experimentally validated, and specific cases of lncRNAs directly regulating SRSF11 are still underexplored in current scientific literature, warranting further investigation.

### Protein-Protein Interactions

SRSF11 interacts with other proteins, such as heterogeneous nuclear ribonucleoproteins (HNRNPs), competitively at alternative splicing sites^12^, ^59^. This competition affects the inclusion or exclusion of exons, ultimately leading to the production of specific splice isoforms^60^. JMJD6 has been shown to regulate the splicing process by specifically binding to the RS domain of SRSF11^28^, ^61^. JMJD6 may further influence splicing regulation through interactions with the U2AF65/U2AF35 complex and SRSF11, suggesting a potential role in coordinating spliceosome assembly and alternative splicing events^61^. However, the precise mechanism by which JMJD6 regulates SRSF11 remains unclear. Further studies are needed to elucidate this regulatory process's pathways and molecular interactions. Such insights could significantly enhance our understanding of SRSF11's role in alternative splicing and its broader implications in cellular functions and disease progression.

### Epigenetic Regulation

Epigenetic modifications, including DNA methylation and histone modifications in the promoter region of the SRSF11 gene, can regulate its expression, particularly in cancer and other diseases^4^, ^62^. For example, METTL3, an m6A methyltransferase, may influence SRSF11 expression indirectly through miRNAs targeting METTL3^63^. METTL3 may also directly modify m6A sites on SRSF11 mRNA, potentially influencing its stability, transport, and translation^33^. However, evidence supporting methylation-based regulation of SRSF11 remains limited. Studies in HCT116 colorectal cancer cells have shown that partial epithelial-mesenchymal transition (EMT) can occur when DNA methyltransferases DNMT1 and DNMT3b are inhibited, leading to selective splicing of the CD44 transmembrane receptor^64^, ^65^. Furthermore, experiments indicate that substances such as alcohol and cocaine can alter the expression of splicing factors, including HSPA6, PCBP1, PTBP1, and SRSF11, by inducing splicing changes through epigenetic modifications^21^, ^40^. For instance, exposure to cocaine has been significantly associated with changes in histone H3 lysine 36 trimethylation levels, resulting in splicing alterations^40^. Cocaine can enrich histone modification H3K36me3 at SRSF11 splicing sites, affecting selective splicing of SRSF11 and subsequently influencing downstream NAc transcripts^21^. These changes lead to widespread differential splicing events in the cerebral cortex, impacting neurobiology and substance use disorders.

## Dysregulation of SRSF11 in Cancer

Aberrant alternative splicing is pivotal in numerous biological processes associated with cancer initiation and progression. These include EMT, apoptosis, cell cycle regulation, proliferation, metabolism, stress response, immune evasion signaling, and invasion^64^-^67^. Dysregulation of SRSF11 has emerged as a critical factor in the pathogenesis of multiple cancers^22^-^24^. By disrupting regular splicing programs and inducing transcriptomic instability, abnormal SRSF11 expression and activity drive carcinogenesis, tumor progression, and therapy resistance. This section explores the mechanisms underlying SRSF11 dysregulation and its impact on cancer-associated pathways (Table 1).

### Breast Cancer

Studies report that the role of SRSF11 is contradictory. Listerman^68^ indicated increases in the levels of β-deletion splice variants of hTERT mRNA, suggesting its role in telomerase activity regulation and promoting papilloma-like breast cancer. Conversely, other Studies show that SRSF11 acts as a tumor suppressor in breast cancer. J. OH^33^ reported that the decreased METTL3 expression leads to reduced SRSF11 expression in breast cancer (BRCA), Colon adenocarcinoma (COAD), lung adenocarcinoma (LUAD), and stomach adenocarcinomas (STAD). Low SRSF11 expression correlates with poor survival and enrichment of p53/apoptosis, inflammation/immune responses, and UV/reactive oxygen species pathways. METTL3 may regulate m6A-modified mRNA splicing through SRSF11, impacting prognosis^63^. Dawid Walerych^69^ found that in breast cancer, knocking down mutant p53 or proteasome activity leads to an increase in SRSF11 levels, suggesting that the mutant p53-proteasome axis may downregulate the expression or stability of SRSF11 through a specific mechanism. However, the exact regulatory mechanism of SRSF11 in breast cancer remains unclear and requires further investigation.

### Colorectal Cancer

In CRC, SRSF11 is overexpressed and associates with poor prognosis^22^. Oncogenic PAK5 phosphorylates SRSF11 at Ser287, preventing ubiquitin-mediated degradation and stabilizing SRSF11^22^. Stabilized SRSF11 drives a pro-metastatic splicing program, including HSPA12A isoform switching, which aligns with increased metastatic potential, reduced survival, and inferior responses to FOLFOX; accordingly, SRSF11 shows promise as a predictive biomarker for FOLFOX benefit^70^.

### Endometrial Cancer

SRSF11 expression is downregulated in endometrial cancer, potentially due to dysregulated HnRNP expression. Further research is needed to elucidate its exact role and clinical significance^71^. These observations are derived primarily from bioinformatics datasets (e.g., TCGA) and lack direct functional validation. Therefore, the relationship between SRSF11 expression, alternative-splicing control, and clinical outcome in endometrial carcinoma should be considered preliminary and hypothesis-generating, warranting further experimental confirmation.

### Gastric Cancer

SRSF11 is significantly upregulated in gastric cancer tissues compared to adjacent normal tissues and is linked to lymph node metastasis and TP53 mutations^23^. High SRSF11 expression across different stages of lymph node metastasis predicts poor prognosis, and its levels correlate with an increased risk of cancer-specific mortality as lymph node metastases increase^23^. SRSF11 may act as an oncogene, facilitating tumor progression and serving as a potential prognostic marker^63^. Elevated SRSF11 expression in gastric cancer also impacts immune cell infiltration, with high macrophage infiltration levels associated with poor outcomes^63^.

### Gliomas

SRSF11 expression correlates with mRNA and protein levels in pediatric central nervous system tumors. Differential expression analysis of known splicing factors in 128 high-grade glioma (HGG) and low-grade samples from 195 patients identified significantly elevated SRSF11 expression in high-grade cases^47^.

### Head and Neck Squamous Cell Carcinoma (HNSCC)

By screening RNA-binding proteins (RBPs) with differential expression between tumor and normal tissues, six candidate genes, including SRSF11, were identified^72^. Prognostic models indicate that SRSF11 overexpression correlates with survival rates in HNSCC patients, highlighting its potential as a prognostic biomarker^73^.

### Hematologic Cancers

In myelodysplastic syndromes (MDS), high SRSF11 expression enhances its recruitment function while reducing the antagonistic effects of splicing suppressors, thereby promoting disease progression^29^. SRSF11 expression is also elevated in multiple myeloma^30^. High SRSF11 expression was found in Acute Myeloid Leukemia(AML), for abnormal telomere length and Htert upregulation promote survival and proliferation of malignant cells^74^, ^75^. However, its precise role in this malignancy remains unclear, highlighting the need for further investigation.

### Liver Cancer

Key case — HCC. *SRSF11* is overexpressed in HCC and exerts pro-tumorigenic functions. Gain- and loss-of-function studies indicate that SRSF11 enhances proliferation and metastatic behavior, whereas its knockdown suppresses growth, at least in part by downregulating CDK1 and G₂/M checkpoint effectors—a cell cycle-centric mechanism^4^, ^20^, ^76^. Additional reports suggest SRSF11 may interface with telomerase engagement, linking RNA-processing programs to replicative immortality, potentially via the TERT/TERC axis, thereby reinforcing its role in disease progression.

Mechanistic tension. Despite these findings, cohort-level associations between SRSF11 expression and clinical outcomes are not consistently robust across HCC subgroups. Correlations between SRSF11 and CDK1 mRNA also appear weak or context-specific. This inconsistency suggests that SRSF11's downstream program may be gated by upstream modifiers such as SRPK/CLK-dependent phosphorylation^14^, ^54^, ^77^, METTL3/m⁶A coupling via RNA methylation regulators, viral etiology (HBV/HCV), and fibrosis stage^4^. These factors likely bias splicing output toward distinct modules—cell cycle versus telomerase—across patient subsets. Notably, the current evidence base is also limited by a lack of stratified validation in large cohorts and methodological heterogeneity across studies.

Working model & testable implications. We propose a context-dependent splicing model in which upstream cues determine whether SRSF11 predominantly drives a CDK1-centric cell-cycle module or a telomerase module in HCC. This model suggests several practical strategies: (i) composite biomarker panels combining SRSF11 × CDK1 ± TERT, rather than single markers; (ii) perturbation studies using SRPK/CLK inhibitors or splice-switching oligonucleotides to test pathway dominance in defined molecular backgrounds; and (iii) etiologic stratification (HBV/HCV/NASH; cirrhosis) in clinical analyses to reconcile dataset disparities and refine prognostic value.

### Oral Cancer

In late-stage oral cancer, the expression levels of SRSF1, SRSF3, SRSF7, SRSF9, SRSF10, and SRSF11 are significantly upregulated compared to the control^24^. SRSF3, SRSF10, and SRSF11 show increased expression as the disease progresses, indicating their involvement in oral cancer progression^24^. SRSF11 overexpression may result from gene amplification or dysregulation of upstream regulatory pathways, such as transcription factors or signaling cascades^78^.

### Ovarian Cancer

The role of SRSF11 in ovarian cancer prognosis remains controversial. Some studies report its overexpression in ovarian cancer tissues, linking it to poor prognosis by promoting cancer cell proliferation, migration, and invasion^79^. Several splicing factors may play key roles in ovarian cancer progression, including SPEN, SF3B5, RNPC3, LUC7L3, SRSF11, and PRPF38B^80^. However, other studies indicate no significant correlation between SRSF11 expression levels and prognosis in ovarian cancer^81^, ^82^. These inconsistent findings underscore that current evidence is largely bioinformatics-based and lacks mechanistic validation, indicating that the proposed oncogenic role of SRSF11 in ovarian cancer remains preliminary.

### Prostate Cancer

RNA sequencing (RNA-seq) analysis of prostate cancer samples from an Indian cohort revealed elevated SRSF11 expression among differentially expressed genes (DEGs)^83^.

### Abnormal Changes in Post-Translational Modifications

Phosphorylation changes in the serine/arginine-rich domain of SRSF11 can affect its activity, subcellular localization, and interactions with the spliceosome^25^. SRSF11 influences the metastatic potential of CRC by regulating alternative splicing of HSPA12A pre-mRNA^22^. Additionally, the oncogenic kinase PAK5 phosphorylates serine 287 of SRSF11, protecting it from ubiquitin-mediated degradation^22^. Cancer-specific post-translational modification patterns may enhance the ability of SRSF11 to drive oncogenic splicing programs, promoting malignancy.

Viruses, as major oncogenic factors, can regulate SRPK-mediated phosphorylation of SRSF11 within cells, influencing spliceosome assembly and the selection of splicing events^31^, ^84^. This, in turn, impacts the splicing patterns of both viral and host mRNAs, regulating viral gene expression^85^. For example, HBV enhances SRPK-mediated phosphorylation of HBc and SRSF11, promoting HBV replication^31^. Conversely, HPV1 can inactivate SRPK1 through exon skipping, reducing SRSF11 phosphorylation and inhibiting HPV proliferation^86^.

### Mutations and Splice Variants

Mutations in the SRSF11 gene or components of the splicing machinery can disrupt its normal function^87^. These mutations may lead to a loss of regulatory control or enhanced activity in cancer contexts. Abnormal splicing of SRSF11 itself can produce dysfunctional isoforms with altered activity. For instance, in myelodysplastic syndrome (MDS), abnormal alternative splicing generates multiple variants of SRSF11^44^. Two variants containing intact RRM and RS domains (uc001deu.2 and uc001dev.3) are highly expressed in MDS samples^29^. In contrast, a variant (uc009wbj.1) containing only the RRM domain but lacking the RS protein interaction domain is highly expressed in control samples^29^.

Thus, in MDS cases, SRSF11 functions as a switch, enhancing its AS recruitment capability and promoting abnormal cell proliferation.

### Abnormal Changes in Non-Coding RNA Regulatory Networks

Non-coding RNAs that regulate SRSF11 expression have been dysregulated in specific cancers. These alterations amplify the oncogenic pathways driven by SRSF11.

For instance, hsa-miR-133 has been identified to regulate SRSF11, along with other genes such as ZC3H15, BCLAF1, KTN1, PRPF40A, and GNL2^57^. Using the NB4 cell model, treatment with hsa-miR-133 inhibited cell proliferation in pediatric acute myeloid leukemia^57^. In prostate cancer cells, miR-26a-5p exerts anti-proliferative effects by reducing viability and migration while inducing cell cycle arrest and apoptosis^88^. SRSF11 has been validated as one of the 73 target genes interacting with miR-26a-5p.Furthermore, stable knockout of miR-29b using CRISPR/Cas9 gene editing in HeLa cells resulted in a significant upregulation of SRSF11 levels^89^. This suggests that miR-29b may have a potential direct inhibitory effect on SRSF11.

lncRNAs play critical roles in alternative splicing, acting as participants and regulators^90^. They influence cancer progression by serving as precursors for messenger RNA (mRNA) splice variants or generating abnormal cancer-related splice variants through selective splicing^91^. Additionally, lncRNAs may directly or indirectly regulate downstream target genes' selective splicing events, thereby influencing cancer development^92^. However, there is currently a lack of evidence demonstrating the direct effects of lncRNAs on SRSF11 regulation.

SRSF11's ability to regulate alternative splicing profoundly affects cancer-related genes^9^. Dysregulation of SRSF11 activity alters splicing patterns of key oncogenes and tumor suppressors, generating isoforms that enhance tumor proliferation or lead to the loss of tumor suppressive activity^68^, ^76^. These changes critically impact cancer-related pathways.

## Comparative Context-Dependent Roles of SRSF11 Across Cancers

Although SRSF11 is broadly upregulated in several malignancies, its biological function is not uniform across cancer types. Evidence from colorectal cancer and hepatocellular carcinoma supports a predominantly oncogenic role through stabilization of proliferative splicing programs (e.g., PAK5-SRSF11-HSPA12A pathway in CRC^22^, ^70^ and SRSF11-CDK1-telomerase circuits in HCC^20^, ^76^). In contrast, studies in breast cancer suggest a context-dependent or even tumor-suppressive effect in certain subtypes, largely influenced by METTL3-dependent modulation of SRSF11 expressio^33^, ^63^ and competition with hnRNP family members during hTERT splicing^68^.

These divergent outcomes likely arise from differences in:

### Upstream Regulatory Networks

CRC and HCC feature strong activation of kinases (PAK5, SRPK/CLK)^22^, ^76^, whereas breast cancer shows METTL3-dependent downregulation of SRSF11^33^, ^63^.

### Distinct Splicing Partners and Chromatin Environments

Breast cancer cells may favor hnRNP-dominant splicing contexts, reducing SRSF11-driven oncogenic isoforms^68^.

### Subtype-Specific Genomic Backgrounds

ER-positive breast cancers (ESR1+) and TP53-mutant tumors exhibit regulatory landscapes that shift SRSF11's activity toward apoptosis or differentiation pathways rather than proliferation^33^, ^69^.

### Tissue-Specific Telomerase Regulation

SRSF11 promotes β-deletion hTERT variants in breast cancer, reducing telomerase activity^68^, whereas in HCC and CRC it enhances telomerase recruitment^20^.

Collectively, these observations support a conditional model, in which SRSF11 acts as an oncogenic driver only when upstream signaling and splicing-partner availability align to support pro-proliferative isoform production. This highlights the need for tumor-type and molecular-subtype-specific analyses when evaluating SRSF11 as a biomarker or therapeutic target.

## Cell Cycle and Proliferation

Dysregulation of SRSF11 has been shown to influence the expression and splicing of key cell cycle regulators, including cyclins and cyclin-dependent kinases (CDKs)^93^. In HCC, knockdown of SRSF11 suppresses CDK1 expression, thereby impairing G2/M transition and inhibiting cell proliferation^76^. However, this CDK1 link is currently specific to HCC, and additional studies are needed to determine whether similar mechanisms operate across other cancer types.

Evidence from other malignancies suggests that SRSF11 may regulate cell cycle-related pathways through distinct splicing networks. In colorectal cancer, SRSF11 interacts with PAK5, promoting alternative splicing of HSPA12A, which supports proliferation and invasion^47^. In gastric cancer, METTL3-mediated m6A modification stabilizes SRSF11 mRNA, indirectly enhancing proliferation through aberrant splicing of downstream targets^59^. n gliomas, SRSF11 modulates the splicing of CDC-like kinase 1 (CLK1), a cell cycle-related splicing factor, by promoting exon 4 inclusion that increases protein expression and activity^94^. Targeted modulation of CLK1 through exon four depletion—achieved by either inhibition or morpholino-guided exon skipping—in the KNS-42 cell line significantly reduces cell proliferation and/or survival rates^47^.

Furthermore, SRSF11 contributes to telomerase activation by enhancing the binding affinity of telomerase to telomeres, thereby promoting sustained proliferation in cancer cells^20^, ^36^. Collectively, these findings indicate that SRSF11 regulates tumor proliferation through multiple, cancer-type-specific splicing programs, rather than a single universal mechanism.

### Apoptosis and Survival

Dysregulation of SRSF11 contributes to the production of anti-apoptotic isoforms (e.g., Bcl-xL) while inhibiting pro-apoptotic variants^5^, ^20^. These changes enhance cell survival under stress, including therapeutic interventions.

Splicing alterations in tumor suppressors like TP53 and PTEN generate nonfunctional or truncated protein isoforms, effectively inactivating their tumor-suppressive roles^23^. These changes impair DNA damage responses and apoptotic pathways, enabling malignant transformation.

In breast cancer, the β-deletion splice variant of human hTERT is inverse-correlated with telomerase activity^68^. Basal-like breast cancer cells have low β-deletion variant levels and high telomerase activity, whereas papillary breast cancer cells have high β-deletion variant levels and low telomerase activity^20^. This suggests that β-deletion variants regulate telomerase activity through splicing. SRSF11 overexpression significantly increases β-deletion splice variant mRNA levels, while hnRNPL or hnRNPH2 overexpression reduces β-deletion levels^68^. These findings suggest that SRSF11 and hnRNPH2 compete for binding sites, regulating the inclusion or exclusion of β-deletion sites^95^.

### Metastasis and Invasion

By regulating the splicing of adhesion molecules and cytoskeletal regulators, SRSF11 promotes cancer cells detachment, migration, and invasion^96^. EMT is a key process in cancer cells invasion and metastasis^97^. Reduced expression of SRSF11 may enhance the EMT pathway, thereby facilitating cancer cells invasion and metastasis^98^, ^99^.

Aberrant activity of SRSF11 in cancer frequently results in the upregulation of splice isoforms that support EMT^22^. Lei et al.^100^ SRSF11 and SRSF1 were significantly upregulated in liver cancer tissues and may regulate the migration and metastasis of liver cancer by selectively splicing exon 3 of SRA1-L. SRSF11 may influence the splicing of genes associated with cell invasion and metastasis, such as MMP-2, MMP-9, and VEGF, as well as EMT-related genes, including CD44 and ZEB1^64^, ^101^, ^102^. Alternative splicing of these genes produces isoforms that enhance cell motility and invasiveness, thereby promoting metastasis—one of the leading causes of cancer-related mortality^103^.

## Therapy Resistance

Splicing alterations driven by SRSF11 promote the expression of isoforms associated with drug resistance, reducing the efficacy of chemotherapy and targeted therapies^104^. In HCC, SRSF11 expression levels are associated with HCC cell drug resistance^76^. This may result from SRSF11's influence on drug target expression or function^27^. High SRSF11 expression decreases sensitivity to multiple drugs, including Sorafenib, CDK inhibitors, DNA replication inhibitors, Nucleotide synthesis inhibitors, PI3K/AKT pathway inhibitors, and BRAF-targeted inhibitors^43^, ^76^, ^100^, ^104^. In colorectal cancer, SRSF11 has been identified as a potential biomarker for FOLFOX resistance and therapy^70^. Screening and validation through datasets such as GSE83129, GSE28702, GSE69657, GSE19860, and GSE41568 link SRSF11 expression with metastatic potential and poor survival in CRC patients^22^. In lung cancer, studies suggest that SRSF11 may modulate radio-sensitivity, affecting responses to radiotherapy^12^.

A comparative summary of SRSF11 dysregulation across major cancer types is presented in Table 2, integrating regulation trends, mechanistic pathways, and experimental validation levels to provide a cross-cancer synthesis.

## SRSF11 as a Cancer Biomarker and Therapeutic Target

As summarized in Table 2, SRSF11 displays cancer-type-specific dysregulation with varying degrees of experimental validation. Its altered expression and function in certain malignancies—most notably CRC, HCC, gastric cancer, and glioma—suggest a potential role as a diagnostic, prognostic, and predictive biomarker^24^. In contrast, findings in other tumor types remain correlative or inconsistent, emphasizing the need for further validation before clinical translation.

The dysregulated expression of SRSF11 in various cancers suggests its potential as a diagnostic, prognostic, and predictive biomarker^24^. Its abnormal expression and functional alterations in cancer cells provide new insights into tumor biology and offer opportunities for personalized cancer therapy^105^ (Table 1).

### Diagnostic Potential

Aberrant SRSF11 expression is most consistently observed in HCC, CRC, gastric cancer, and glioma, where upregulation correlates with tumor progression, metastasis, and reduced overall survival^106^. However, findings in ovarian, breast, and prostate cancers remain preliminary and require further validation.

In CRC, high SRSF11 expression enhances metastatic potential and therapy resistance^17^, ^22^. Similarly, in HCC, SRSF11 levels correlate with the expression of cell cycle genes, such as CDK1, further highlighting its importance as an indicator of tumor aggressiveness^76^, ^100^, ^107^. Evidence in oral, HNSCC, and prostate cancers remains preliminary^83^, ^88^, ^108^. Research on gastric cancer has revealed that SRSF11 overexpression in gastric cancer tissues is linked to poor prognosis^33^, ^63^. These data collectively indicate diagnostic potential, though standardized validation is still lacking.

### Assay Feasibility and Multi-Center Validation

At present, no standardized IHC or RT-qPCR protocol exists for reliable SRSF11 quantification. Developing reproducible detection assays will be critical for clinical use. Feasibility studies could employ RNA-seq or circulating-RNA profiling from plasma/serum to detect SRSF11 transcripts^109^, ^110^. Multi-center validation using harmonized extraction, normalization, and quantification methods will be required to establish diagnostic thresholds and inter-laboratory consistency.

### Prognostic Value

The expression levels of SRSF11 have shown prognostic value in several types of cancer. In HNSCC, predictive models based on SRSF11 overexpression have been developed to predict disease progression and patient survival rates, demonstrating its potential as a prognostic indicator^72^. Similarly, studies on oral cancer indicate that SRSF11 expression levels can serve as predictive biomarkers to assess patient survival and recurrence risk^24^, ^111^. However, subtype-specific or contradictory findings in ovarian and breast tumors suggest context-dependent prognostic relevance.

### Integrated Mechanistic and Multi-Marker Biomarker Potential

The biomarker relevance of SRSF11 is reinforced by its molecular mechanisms and by its ability to interact with other oncogenic regulators^112^. Functionally, SRSF11 modulates alternative splicing events that generate oncogenic isoforms affecting cell-cycle genes, apoptosis regulators, EMT mediators, and telomerase components^46^, ^64^, ^113^. Its role as a nuclear speckle-targeting factor further suggests a critical function in telomerase regulation and genome stability, closely linked to cancer development^84^, ^114^. Knockdown of SRSF11 disrupts CDK1 expression, telomere maintenance, and cell-cycle progression^76^, ^115^, confirming its central role in tumor biology. These findings underscore the functional significance of SRSF11 in tumor biology and reinforce its potential application as a biomarker.

Based on these mechanisms, SRSF11 could be integrated into multi-marker diagnostic and prognostic panels alongside key pathway partners such as CDK1 and TERT. A composite SRSF11 × CDK1 × TERT signature may enhance the precision of tumor classification, particularly in HCC and CRC, where these splicing networks converge. Incorporating mechanistically linked markers into multiplex RT-qPCR or RNA-seq-based assays could improve both sensitivity and specificity for early detection and prognostic assessment. Future studies should validate these combined markers in large, clinically annotated cohorts, evaluating their additive predictive value, reproducibility, and clinical utility across cancer subtypes.

### Therapeutic Implications

SRSF11 acts downstream of SRPKs and CLKs, which modulate its phosphorylation and splicing activity. Inhibitors targeting SRPK/CLK signaling are currently in early clinical testing and could indirectly modulate SRSF11-dependent splicing events. Furthermore, splice-switching oligonucleotides (SSOs) may offer a direct strategy to correct aberrant SRSF11-mediated isoforms. These approaches provide promising avenues for the therapeutic modulation of splicing programs in cancer.

## Current Challenges and Knowledge Gaps

Despite growing evidence supporting the role of SRSF11 in cancer, several challenges remain. Its functions in different tissues and cancer types are not fully understood, making it challenging to classify SRSF11 as an oncogene or tumor suppressor.

The regulatory networks and post-translational modifications controlling SRSF11 activity need further exploration. Studies on its prognostic value have shown conflicting results, particularly in breast and ovarian cancers. For instance, some reports suggest SRSF11 promotes tumor growth in breast cancer, while others indicate it may act as a suppressor, depending on the context. Similarly, studies on ovarian cancer show inconsistent associations with prognosis, emphasizing the need for standardized methods and larger sample sizes.

Although SRSF11 shows promise as a biomarker and therapeutic target, its clinical utility is limited by the lack of validated assays to measure its activity and effects on splicing. More research is required to confirm its role as a predictive biomarker for treatment responses, particularly in therapies targeting alternative splicing. Long-term studies correlating SRSF11 expression with treatment outcomes are critical to determining its clinical relevance and potential in personalized therapy.

The influence of non-coding RNAs and epigenetic modifications on SRSF11 regulation is another area that remains underexplored. Comprehensive studies using multi-omics approaches are needed to map its regulatory network and better understand its roles in therapy resistance, metastasis, and immune evasion in cancer. Future applications of emerging technologies, such as genome-wide CRISPR-Cas9 and integrative multi-omics approaches, are expected to uncover the regulatory mechanisms further and signaling pathways of SRSF11. Investigate RNA-targeting therapies, such as antisense oligonucleotides and small-molecule inhibitors, to modify SRSF11 activity. Preclinical trials should evaluate their effectiveness in correcting splicing abnormalities and enhancing treatment responses.

## Conclusion

SRSF11 has emerged as a key splicing regulator with multifaceted but context-dependent roles in tumor biology. It orchestrates aernative-splicing programs that influence cell-cycle progression, telomerase activity, EMT, and immune modulation. However, robust mechanistic evidence currently exists mainly in colorectal, hepatocellular, gastric, and glioma models, while observations in other malignancies remain correlative or preliminary.

Comparative analyses reveal both conserved and cancer-type-specific modules: the CDK1-centered cell-cycle network represents a conserved axis, whereas the PAK5-SRSF11-HSPA12A pathway in colorectal cancer and the CDK1-telomerase modules in HCC exemplify tissue-restricted mechanisms. These findings support a model in which SRSF11 acts as a conditional oncogenic regulator whose impact varies according to upstream signaling context, tumor microenvironment, and splicing-partner availability.

Despite accumulating experimental evidence, important knowledge gaps remain, including incomplete mapping of SRSF11's upstream modifiers, inconsistent cohort-level associations, and limited mechanistic resolution across tumor types. Addressing these gaps will require multi-omics integration, etiologic and stage-specific stratification, and standardized assays for SRSF11 detection and quantification.

Future studies should prioritise composite biomarker panels (e.g., SRSF11×CDK1±TERT), targeted perturbation of splice programs via SRPK/CLK inhibitors or splice-switching oligonucleotides, and systematic evaluation of SRSF11's prognostic utility across etiologic subtypes. Translationally, the context-dependent nature of SRSF11's oncogenic modules offer a conceptual basis for precision therapeutic strategies in splicing-targeted oncology.

In summary, this review integrates current mechanistic knowledge of SRSF11 across cancers, distinguishing confirmed from hypothetical pathways, and proposes a conceptual framework for translating these insights into precision, splicing-based therapeutic strategies.

## Literature search and selection

We conducted a structured literature search of PubMed, Web of Science, and Scopus through July 2025 using the terms “SRSF11” OR “SFRS11” AND (“splicing” OR “RNA processing” OR “cancer”). Only peer-reviewed English-language articles were considered, including both original experimental studies and relevant reviews.

Titles and abstracts were screened to identify publications that investigated SRSF11 expression, regulation, or function in human cancers or experimental models. Reference lists of included papers were also manually reviewed to capture additional eligible studies.

Inclusion criteria emphasized studies providing mechanistic evidence (e.g., gain- or loss-of-function assays, splicing validation), translational endpoints (diagnostic, prognostic, or therapeutic relevance), or multi-omics support. Reports limited to expression correlation without functional verification were marked as preliminary and interpreted cautiously.

Although this review follows a narrative synthesis framework, the search process was systematic and transparent to minimize selection bias. Risk of bias was not formally assessed but considered during interpretation of individual studies.

## Figures and Tables

**Table 1:** Summary of key studies reporting SRSF11 dysregulation in cancer.

Cancer Type	Dysregulation of SRSF11	Mechanism	Biomarker	Reference
Acute Myeloid Leukemia	overexpression	SRSF11 causes abnormal telomere length, and hTERT upregulation promotes the survival and proliferation of malignant cells	candidate diagnosticbiomarker	46, 57
Breast Cancer	overexpression	Alternative splicing to β-deletion splice variants of hTERT.	controversial	68
	downregulation	METTL3 downregulates the expression or stability of SRSF11.		33, 63
Colorectal Cancer	overexpression	PAK5 phosphorylates serine 287 of SRSF11, preventing its ubiquitin-mediated degradation	candidate diagnostic/prognostic biomarker	22, 70
Endometrial Cancer	downregulation	dysregulated HnRNP expression inhibits SRSF11	unknown	71
Gastric Cancer	overexpression	unknown	unknown	23, 63
Glioma	overexpression	SRSF11 promotes CLK1 expression and enhances cell proliferation	candidate diagnostic biomarker	47
Head and Neck Squamous Cell Carcinoma	overexpression	unknown	candidate diagnostic biomarker	72, 73
Liver Cancer	overexpression	SRSF11 promotes CDK1 expression and enhances cell proliferation; reactivation of hTERT drives HCC development.	candidate diagnostic/prognostic biomarker	20, 76
myelodysplastic syndromes	overexpression	abnormal alternative splicing generates multiple variants of SRSF11	candidate diagnostic biomarker	30
myeloid leukemia	overexpression	unknown	unknown	30
Oral Cancer	overexpression	unknown	candidate diagnostic/prognostic biomarker	24, 78
Ovarian Cancer	overexpression	unknown	controversial	79-82
Prostate Cancer	overexpression	unknown	unknown	83

*“Unknown” indicates that SRSF11 dysregulation has been reported but mechanisms remain unvalidated. “Controversial” refers to cancer types with inconsistent or conflicting findings across studies.

**Table 2:** Comparative summary of SRSF11 regulation, mechanistic axes, and evidence levels across cancers.

Cancer Type	Regulation Trend	Key Mechanistic Axis / Pathway	Experimental Evidence	Clinical Endpoint / Observation	Evidence Level
Colorectal cancer (CRC)	Upregulated	PAK5-SRSF11-HSPA12A axis; regulates HSPA12A splicing^47^	In vitro / in vivo validation (knockdown + rescue)	Promotes proliferation, invasion, therapy resistance	Confirmed
Hepatocellular carcinoma (HCC)	Upregulated	SRSF11-CDK1-hTERT axis; regulates cell-cycle and telomerase splicing^76^	siRNA knockdown, RT-qPCR, WB, tumor model	Enhances proliferation and telomerase activity	Confirmed
Gastric cancer	Upregulated	METTL3-SRSF11-m6A pathway^59^	METTL3 overexpression stabilizes SRSF11	Facilitates proliferation and migration	Suggestive
Glioma	Upregulated	SRSF11-CLK1 splicing modulation^47^	Morpholino exon-skipping assay	Alters cell-cycle progression and survival	Suggestive
Ovarian cancer	Variable	Correlative changes in splicing networks^77^	RNA-seq and bioinformatics data	Correlation with late-stage disease	Preliminary
Breast cancer	Variable	Indirect correlation with METTL3 expression^59^	Transcriptomic analyses only	Prognostic trends inconsistent	Preliminary
Prostate cancer	Unclear	Predicted SRSF11-related isoforms^82^	TCGA correlation only	No validated clinical association	Preliminary

## Abbreviations

SRSF11: Serine/arginine-rich splicing factor 11
EMT: epithelial-mesenchymal transition
pre-mRNAs: Precursor messenger RNAs
CRC: colorectal cancer
HCC: hepatocellular carcinoma
AS: alternative splicing
RRM: RNA recognition motif
AA: amino acids
ESE: exonic splicing enhancer
ES: exon skipping
IR: intron retention
MXE: mutually exclusive exons
TERC: telomerase RNA component
PTMs: post-translational modifications
hTERT: human telomerase reverse transcriptase
SRPKs: SR protein kinases
CLKs: Cdc2-like kinases
TRN-SR2: transportin-SR2
HSPCs: hematopoietic stem and progenitor cells
ncRNAs: non-coding RNAs
snoRNAs: small nucleolar RNAs
miRNAs: microRNAs
lncRNAs: long non-coding RNAs
pAML: primary acute myeloid leukemia
GTB: gradient boosting tree
HNRNPs: heterogeneous nuclear ribonucleoproteins
BRCA: breast cancer
COAD: Colon adenocarcinoma
LUAD: lung adenocarcinoma
STAD: stomach adenocarcinomas
HGG: high-grade glioma
HNSCC: Head and Neck Squamous Cell Carcinoma
RBPs: RNA-binding proteins
MDS: myelodysplastic syndromes
AML: Acute Myeloid Leukemia
RNA-seq: RNA sequencing
DEGs: differentially expressed genes
ESR1+: ER-positive breast cancers
CDKs: cyclin-dependent kinases
CLK1: CDC-like kinase 1
